# Adhesive bowel obstruction? Not always

**DOI:** 10.4103/0974-2700.76826

**Published:** 2011

**Authors:** D Mittapalli, B J Sebastian, E Leung, N Barnes, P S P Senapati

**Affiliations:** 1 Department of Surgery, Rochdale Infirmary, Whitehall Street, Rochdale, OL12 0NB, UK; 2 Manor Hospital, Moat Road, Walsall, WS2 9XS, UK

**Keywords:** Adhesion, intestinal lymphoma, Non-Hodgkins lymphoma, small bowel obstruction

## Abstract

A 58-year-old man presented acutely with features of post-surgical adhesive small bowel obstruction. Following an unsuccessful trial of conservative management, computed tomography (CT) of the abdomen was performed. This revealed a mass in the ileocaecal region, for which he underwent a subsequent right hemicolectomy. Histology revealed diffuse B-cell Non-Hodgkin’s lymphoma of the terminal ileum. Confounding obstructive lesion of the intestine in patients with a history of previous laparotomy is extremely uncommon. Early high resolution imaging may predict diagnosis and consolidate clinical management plans.

## INTRODUCTION

Adhesions after abdominal surgery are so common that often no other diagnosis is considered in patients presenting with symptoms and signs consistent with SBO: dilated small bowel loops on plain abdominal X-ray and abdominal scar(s). Intra-abdominal adhesions are fibrous bands of scar tissue that bind two separate parts of tissue together. These adhesions develop when the body’s repair mechanisms respond to tissue disturbance, such as infection, trauma, radiation and surgery. It is reported that 93% of patients develop adhesions after one laparotomy compared to 10% of patients with no previous abdominal surgery.[1] Fortunately, less than 1% develop SBO within one year of laparotomy. Moreover, these patients have equal risk of developing another pathology as the general population.

Other common causes of SBO include tumour of the caecum, lesions within the small bowel and extrinsic compression by intersussception, hernial neck or extraintestinal tumours. We report a case of a 58 year-old Asian man, with a history of laparotomy for perforated appendicitis a few years earlier, who presented acutely with SBO. Eventually, it was found the cause be due to lymphoma of the terminal ileum. We present the case, discuss management and review the literature.

## CASE REPORT

**Figure 1:**
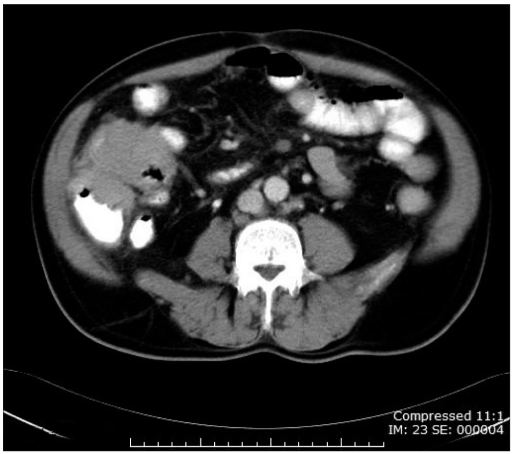
CT scan demonstrating a cecal mass

A 58-year-old man presented acutely with clinical and radiological (plain abdominal X-rays) features of SBO. There was no significant past medical history. Past surgical history included cholecystectomy and emergency laparotomy for perforated appendicitis, which were performed years ago. On physical examination, he was hemodynamically stable. His abdomen was diffusely distended and tender, with no signs of peritoneal irritation. There were no palpable masses and bowel sounds were high-pitched. No external hernias were identified. All laboratory investigations were within normal limits, including hemoglobin concentration. Given that, adhesions were considered the most likely cause. Following an unsuccessful trial of conservative management including nasogastric aspiration and intravenous fluids for 48 hours; contrasted abdominal CT was performed on day 3. This revealed a mass in the ileocecal region [Figure 1], for which he subsequently underwent right hemicolectomy. Histology revealed diffuse B-cell Non-Hodgkin’s lymphoma of the terminal ileum. The patient was referred for chemotherapy. Four years on, he remained disease-free.

Adhesive SBO accounts for about 1% of all emergency surgical admissions and 3.3% of all emergency laparotomies.[1] It can occur any time following initial surgery.[2] While management of uncomplicated adhesive SBO is largely conservative, the debate remains over the optimal duration for such non-operative management[3] since only 8% of cases require surgical intervention. Confounding obstructive lesion of the intestine in patients with previous laparotomy is extremely uncommon. As adhesive SBO was the most likely diagnosis, our patient’s initial management was considered acceptable. Early high-resolution imaging may elucidate etiology and consolidate clinical management plans.[4] In our patient’s case, early CT scan would have led to immediate laparotomy without delay. Therefore, if surgical intervention is deferred on clinical grounds, CT scan should be performed as soon as practicable, provided that there are no contraindications.
